# Early Fall in C-Reactive Protein (CRP) Level Predicts Response to Tocilizumab in Rapidly Progressing COVID-19: Experience in a Single-Arm Pakistani Center

**DOI:** 10.7759/cureus.20031

**Published:** 2021-11-30

**Authors:** Saba Khurshid, Neelum Rehman, Saad Ahmed, Bilal Ahmad, Mustafa Khurshid, Anjum Muhammad, Fuad A Siddiqi, Dure Nayab, Hasan Saleem, Zulqurnain Saleem

**Affiliations:** 1 Medicine, Khyber Medical University, Peshawar, PAK; 2 Medicine, Pak-Emirates Military Hospital, Rawalpindi, PAK; 3 Endocrinology and Diabetes, Pak-Emirates Military Hospital, Rawalpindi, PAK; 4 Medicine, Rehman Medical Institute, Peshawar, PAK; 5 Dermatology, Pak-Emirates Military Hospital, Rawalpindi, PAK; 6 Medicine, Combined Military Hospital, Rawalpindi, PAK; 7 Pediatrics, Medics Hospital Rawalpindi, Rawalpindi, PAK

**Keywords:** survival outcome covid-19, covid-19 treatment, crp, tocilizumab, covid-19

## Abstract

Introduction

There are conflicting studies regarding the efficacy of tocilizumab use in coronavirus disease 2019 (COVID-19) disease. There is a special need to identify the parameters that could predict its response in early COVID-19 disease.

Objective

To report our experience with tocilizumab and correlate the magnitude of fall in c-reactive protein (CRP) as a predictor of its response to treatment in early COVID-19 disease.

Methods

All confirmed COVID-19 cases admitted to a tertiary healthcare hospital in Peshawar Pakistan, receiving ≥1 dose of intravenous tocilizumab, between March and September 2020 were included. Relevant clinical data of the patients were recorded and further divided into two categories based on the relative fall in CRP levels, 48 hours after tocilizumab administration. Adequate response (≥50% fall from baseline CRP), primary outcomes (fall in oxygen requirement and inflammatory biomarkers), and secondary outcome (all-cause mortality at day 28) were recorded. All outcomes were compared based on falls in CRP levels.

Results

A total of 27 patients were included. Males were 24 (88.8%) while females were three (11.1%). The mean age was 60.9±11.6 years. The mean day of illness at the time of tocilizumab administration was 4.26±3 days. After 48 hours of tocilizumab administration, 17 (62.9%) patients showed clinical improvement, with the mean SaO_2_/FiO_2 _ratio prior to treatment significantly increased (p<0.01). A significant reduction in CRP and ferritin levels was seen post-treatment (p <0.01 and p<0.01, respectively). Twenty (74.1%) patients demonstrated adequate response to tocilizumab while seven (25.9%) showed an inadequate response. Patients with adequate response had higher chances of improvement in oxygenation and lower in-hospital mortality (*p-*value 0.009 and 0.020, respectively).

Conclusions

Tocilizumab shows clinical improvement in a vast majority of patients. Being an early and sensitive predictor, a fall of ≥50% in CRP at 48 hours can be used to predict the overall response to tocilizumab as a guide to treatment.

## Introduction

First discovered in Wuhan, China, in December 2019, coronavirus disease 2019 (COVID-19) caused by severe acute respiratory syndrome coronavirus 2 (SARS-CoV-2) has rapidly spread across the planet. The spectrum of illnesses from COVID-19 is highly heterogeneous, ranging from mild subclinical infection to respiratory failure, acute respiratory distress syndrome (ARDS), and death.

Many pathological mechanisms for disease progression have been postulated. The cytokine storm and associated hyper-inflammation have been shown to play a central role in the rapid progression of the disease. This is supported further by the rapid rise in interleukin-6 (IL-6) and c-reactive protein (CRP) levels seen in severe and rapidly deteriorating patients [1-2].

Various treatment modalities have been tested with different rationales and results. Truncating the inflammatory response early in its course and preventing significant lung damage is a well-recognized target of the treatment modalities.

Tocilizumab, a monoclonal antibody that binds to and blocks the IL-6 receptor-mediated inflammatory response is approved for the treatment of rheumatologic disorders and cytokine-release syndrome. Initially, many observational studies demonstrated good outcomes with tocilizumab, and its off-label and compassionate use in cytokine storms became widespread [3-6]. Results from high-power randomized control trials have been conflicting. Where trials like EMPACTA (Evaluating Minority Patients with Actemra) and REMCAP (Randomised, Embedded, Multi-factorial, Adaptive Platform Trial) have reported benefits in mortality and clinical conditions, others reported no clear benefit [7]. Tocilizumab is recommended by both the Infectious Diseases Society of America (IDSA) [8] and the National Institutes of Health (NIH) guidelines in rapidly deteriorating patients.

Very little local data from Pakistan are available on the use of tocilizumab. We aimed to add to the existing literature by describing our experience of tocilizumab use in patients undergoing an active cytokine storm and rapid clinical deterioration. Owing to the variable results and uncertainty surrounding tocilizumab use, there is a need to assess and predict the response to treatment early in the disease. As a readily available and sensitive biomarker of severe COVID-19 disease, we studied the magnitude of fall in CRP as a predictor of response to tocilizumab treatment.

## Materials and methods

This retrospective observational study was conducted at a tertiary healthcare setup from March to September 2020. Approval from the institutional ethical committee, Khyber Teaching Hospital, Khyber Medical University, was obtained (IRB number: 986/DME/KMC). All positive COVID-19 PCR or high-resolution computed tomography (HRCT) patients, who received one or more doses of intravenous tocilizumab (8 mg/kg rounded to the nearest vial size, as per institution protocol) were included. Patient selection for tocilizumab administration was based on rapid clinical deterioration/rise in oxygen requirement and/or severe derangement in inflammatory biomarkers, signaling the onset of cytokine release syndrome. All patients received a single dose of 8 mg/kg rounded to the nearest vial size as per institution protocol. The data were collected from a review of patient hospital records. Information regarding patients’ demographic characteristics (age, gender, comorbid conditions), duration of symptoms prior to tocilizumab administration, length of hospital stay, and outcome at day 28 after receiving tocilizumab were recorded. Disease severity, oxygen requirement, vital signs, including oxygen saturation (SpO2), and laboratory parameters, including inflammatory markers (CRP, ferritin, D-dimers) were recorded up to 24 hours prior and 48 hours after tocilizumab administration. The clinical condition was recorded on a seven-point ordinal scale with scoring assigned as 1=Room air and not hospitalized, 2=Hospitalized but not on supplemental oxygen, 3=Receiving low flow supplemental oxygen, 4=Receiving high-flow supplemental oxygen, 5=On non-invasive ventilation, 6=On invasive mechanical ventilation, and 7=Death. Oxygen requirement was assessed using SpO2/fraction of inspired oxygen (FiO2) ratio, a reliable surrogate for partial pressure of oxygen (PaO2)/FiO2 ratio. Patients were further divided into two categories based on relative fall in CRP levels, observed 48 hours after tocilizumab administration. The adequate response was defined as a 50% or higher decrease in CRP levels from baseline at 48 hours following tocilizumab administration. Primary outcomes were defined as improvement in clinical condition, decrease in oxygen requirement, and fall in inflammatory biomarkers 48 hours after tocilizumab administration. All-cause mortality at day 28 was observed as a secondary outcome. Improvement in clinical condition was defined as an improvement by one or more points in the oxygen support category at 48 hours post tocilizumab administration.

All outcomes were compared further between patients demonstrating adequate response to those not demonstrating adequate response based on falls in CRP levels.

For statistical analysis, categorical variables are presented as frequencies and percentages while continuous variables are presented as means with standard deviations for normally distributed variables and medians with interquartile ranges for non-normally distributed variables. Between groups, comparisons were performed using the Fisher exact test for categorical variables, the student's t-test for continuous normally distributed variables, and the Mann-Whitney U test for non-parametric continuous variables. Pre and post-treatment values were compared using the Wilcoxon signed-rank test. The Kolmogorov-Smirnov test was used to assess variables for normality. A p-value of <0.05 was regarded as statistically significant.

## Results

A total of 27 patients were included in our study. Males accounted for 88.8% (n=24) of the patients while females were 11.1% (n=3). The mean age of patients was 60.9±11.6 years. The mean day of illness at the time of tocilizumab administration was 4.26±3.08 days. Diabetes (48.1%, n=13) was the most prevalent comorbid condition, followed by hypertension (40.7%, n=11).

At baseline prior to tocilizumab administration, 59.3% (n=16) patients were on high-flow oxygen (category 3), 18.5% (n=5) on low-flow (category 2), 11.1 %( n=3) were receiving non-invasive ventilation, 7.4 %( n=2) patients were on invasive mechanical ventilation (category 5), while 3.7% (n=1) patients were breathing ambient room air. After 48 hours of tocilizumab administration, 62.9% (n=17) of patients showed clinical improvement, eight patients remained at the same level of oxygen support while two patients underwent clinical worsening. The mean SaO2/FiO2 ratio of patients prior to treatment was 128.8±66.4, which increased to 300.6±160.8 48 hours after treatment with a p-value of <0.01. Details of oxygen support category before and after treatment are listed in Table 1.

**Table 1:** Details of oxygen support categories before and after treatment CRP: c-reactive protein; SpO2: oxygen saturation; FiO2: fraction of inspired oxygen

Parameter	Pre-treatment	Post-treatment (after 48 hours)	p-value
CRP	144.8±117.3	33.2±52.7	<0.01
D dimer	1797.0±1426	1907.1±2396.6	0.799
Ferritin	1523±923.6	1064.37	0.045
Respiratory rate	37.9±7.7	22.5±8.8	0.02
SpO2	87.4±5.8	92.3±4.7	0.02
FiO2	75.4±17.8	46.3±31.8	<0.01
S/F ratio	128.8±66.4	300.6±160.8	<0.01

Pre-treatment CRP (144.8±117.3) and ferritin (1523±923.6) levels were significantly higher compared to post-treatment (CRP: 33.2±52.7, p <0.01; ferritin 1064±600, p-value 0.045). There was a slight decrease in the neutrophil-lymphocyte ratio and a slight increase in D-dimer levels following treatment, however, these were statistically insignificant.

On the 28th day, 22.2% (n=6) of patients had died. Seventeen (62.9%) were successfully discharged home while 15% (n=4) remained admitted to the hospital on day 28.

Overall, 74.1% (n=20) patients demonstrated adequate response to tocilizumab based on CRP fall compared to 25.9% (n=7) patients who showed inadequate response. A detailed comparison of baseline demographics, outcomes, and pre and post-treatment inflammatory markers stratified based on response to treatment is listed in Table 2.

**Table 2:** A detailed comparison of baseline demographics, outcomes, and pre and post-treatment inflammatory markers stratified based on response to treatment CRP: c-reactive protein; COPD: chronic inflammatory lung disease; AKI: acute kidney injury

Parameter	Responders n=20	Non-Responders	p-value
Age	57.6±10.6	70.3±9.1	0.010
Male, Female	17 (%), 3(%)	7 (%), 0 (0%)	0.277
Diabetes mellitus	9 (45%)	4 (57.1%)	0.580
Hypertension	7 (35%)	4 (57.1%)	0.305
COPD	0 (0%)	2 (28.6%)	0.013
Asthma	1 (5%)	2 (28.6%)	0.088
Smoking	1 (5%)	0 (0%)	0.547
Obesity	1 (5%)	0 (0%)	0.547
AKI	0 (0%)	1 (14.3%)	0.085
Ischemic heart disease	1 (5%)	1 (14.3%)	0.419
Duration of symptoms on the day of tocilizumab administration	3.78±3.5	1.17±0.4	0.087
Deaths	2 (10%)	4 (57.1%)	0.020
CRP pre-treatment	163.5±118.8	91.4±102.3	0.145
CRP post-treatment	16.0±17.1	82.5±85.5	0.162
Ferritin pre-treatment	1398±658	1879±1468	0.698
Ferritin post-treatment	954±561	1379±719	0.143
D-dimer pre-treatment	1477±1131	2712±1857	0.072
D-dimer post-treatment	1662±2497	2607±2093	0.043
Neutrophil-lymphocyte ratio, pre-treatment	9.1±4.0	5.8±2.9	0.041
Neutrophil-lymphocyte ratio, post-treatment	7.53±5.30	6.75±3.25	0.946
Clinical improvement	16 (80%)	1 (14.3%)	0.009
S/F ratio pre-treatment	135.0±76.1	110.9±16.2	0.685
S/F ratio post-treatment	319.1±154.3	247.6±179.3	0.145
HB pre-treatment	12.5±1.8	13.8±2.4	0.263
HB post-treatment	11.9±2.1	12.9±1.25	0.314
Platelets pre-treatment	217.6±74.4	239.3±88.9	0.850
Platelets post-treatment	241.5±77.2	201.7±53.7	0.240
Total leucocyte count pre-treatment	9.7±3.8	6.7±2.4	0.072
Total leucocyte count post-treatment	10.2±4.1	9.5±5.0	0.935

Both subgroups did not differ significantly in baseline demographics and pre-treatment laboratory parameters. However, patients with adequate response after administration had higher chances of improvement in oxygenation (OR: 24.0 [2.213 - 260.286] p=0.009) and lower in-hospital mortality (OR=0.83 [0.010 -0.675] p=.020). The mean SF ratio after treatment was higher in patients with an adequate response (319.1±154.3) compared to those without adequate response (247.5±179.4); however, this difference was statistically insignificant (p=0.145).

## Discussion

Our study demonstrated that tocilizumab showed improvement in various clinical as well as laboratory parameters in a vast majority of our cohort. An improvement was demonstrated in 62.9% of the patients. Comparable results were reported in various observational studies. Mushtaq et al., in their single-arm analysis, reported that 77.5% had an improvement in oxygen requirement [9]. Similarly, Knorr et al. in their study reported that 40.9% of patients had a one or more points improvement on an ordinal clinical scale on day 7 [10]. Xu et al. also showed a decreased oxygen requirement in 75% of patients following Tocilizumab administration [11]. Although an improvement in clinical status has been reported consistently in many observational studies, this effect has not been demonstrated in many RCTs. The COVACTA trial with 294 patients showed no overall difference in clinical status after 28 days compared to placebo [7]. Similarly, the BACC Bay trial (Boston Area COVID-19 Consortium trial) did not demonstrate any improvement in intubation or death, and, in fact, saw 17% of patients worsening clinically [7]. In contrast, the CORIMUNO and REMAP CAP (Randomized Embedded Multifactorial Adaptive Platform for Community-Acquired Pneumonia) trials did show improved ventilator-free survival after tocilizumab use [12-13]. Since we assessed response relatively earlier, i.e. after 48 hours, it was pertinent to use a one-point improvement as a marker of clinical response as opposed to most studies that defined it as a two-point improvement.

The timing of administration has been a long subject of debate in tocilizumab use, with early administration showing better outcomes [14-16]. Although the study period was earlier than when guidelines were issued by IDSA18 for tocilizumab administration, which recommends a single dose for patients rapidly deteriorating in terms of oxygen requirement, it is interesting to note that a majority of our patients did receive tocilizumab within the recommended time frame.

As with previous studies, the pre-treatment level of inflammatory markers negatively correlated with both clinical improvement and patient outcomes, suggesting that those with the severe underlying disease may be less amenable to treatment with tocilizumab, in line with the suggestion that the aim is to abut the cytokine hyper-inflammatory response earlier, preventing damage to the lungs, with limited role once the damage has already occurred.

Overall mortality from our study is similar to the pooled mortality rate of 19% reported by O. Berardicurti et al. [17] in their systematic review and meta-analysis. This is comparable to observational studies, with Morena et al. reporting 27%, Toniati et al. 20%, and Hill et al. 21%. Alattar et al. reported a slightly lower mortality rate of 12% [18-21]. Studies from Pakistan have shown similar results. with Nasir et al. reporting overall mortality of 23% and Mushtaq et al, 22.5%9 [9,22]. Interestingly, the mortality rate reported in the tocilizumab arms of several randomized controlled clinical trials is highly variable and many do not report a statistically significant improvement in mortality compared to placebo. The RECOVERY trial was the first trial to report a significant decrease in mortality in patients treated with tocilizumab [23]. The REMAP-CAP trial showed a mortality of 28% in the tocilizumab arm and overall improved days off oxygen support [13]. However, the CORIMUNO, TOCIBRAS, and COVACTA trials showed no mortality benefit of tocilizumab use [14,24-25].

Another aspect that may contribute to the better response seen in our study is that all patients received a low-dose corticosteroid, 6 mg dexamethasone, as the standard of care. Multiple studies have studied the combined effect of tocilizumab and corticosteroids and shown that the combination is shown to improve mortality than either drug alone [26-27]. Since all patients received the same dose of corticosteroids early in the disease, the compounded benefit of steroids in addition to tocilizumab in our study cannot be ruled out.

The fall in inflammatory biomarkers has been well-studied and documented in almost all studies on tocilizumab and corticosteroids [9,28]. This effect on CRP is not limited to and is in line with the mechanism of tocilizumab [28], as IL-6 is shown to induce synthesis of acute-phase reactants in the liver, and hence blockage of this pathway would lead to a fall in observed levels. The blockade of IL-6 activity with tocilizumab can therefore improve CRP levels by both decreasing the synthesis of CRP, as well as an overall clinical improvement and decreased inflammation. We hypothesize that this fall in CRP can be used to predict the overall response to tocilizumab. Considering conflicting results from different studies, we believe an early and sensitive predictor can be used to guide treatment. Our results indicate that a 50% fall at 48 hours significantly predicts response to treatment with tocilizumab. A similar effect of fall in CRP was studied by Cui et al. with the use of dexamethasone [29]. Their results revealed that a 50% fall 48-72 hours after dexamethasone initiation is predictive of good response and lower mortality in patients. Since all of our patients had already received corticosteroids as part of the standard of care for over 48 hours, this could explain that all our patients were already in the bracket not fully responding, both clinically and in terms of laboratory values, to steroids. Our results go beyond this and use the level of CRP to predict the adequate response to tocilizumab, especially in those not responding adequately to corticosteroids. In light of mixed results reported in studies, it is pertinent to ask whether all patients respond similarly to tocilizumab, and we believe our results are in line that an early assessment of response to tocilizumab and seeking alternate or escalated treatment in unresponsive patients warrants further study. Whether this inadequate response is due to a relative under-dosing due to a relatively more severe cytokine syndrome (check for differences in baseline characteristics of responders and non-responders) or due to patients having variable underlying mechanisms for deterioration cannot be reasonably concluded.

Our study has some limitations. First, the observational design and the lack of a control group. Second, the small sample size. In addition, the patient’s laboratory parameters were not followed beyond 48 hours so details of patterns beyond the immediate period, in line with the long elimination half-life of tocilizumab, cannot be established. With only three in total, women are underrepresented in our study. This may be, in part, due to the overall small sample size; however, most studies have reported more men admitted than women and this may be a continuation/exaggeration of the same effect.

## Conclusions

With improvement demonstrated in the various clinical parameters in 62.9%, tocilizumab may show clinical betterment in a vast majority of patients. However, the possibility that this better response to tocilizumab may have been seen when the drug is compounded with corticosteroids cannot be ruled out. These results are comparable in various observational studies. The higher the pre-treatment level of inflammatory markers, the lower the chance of the underlying severe disease to be amenable to treatment with tocilizumab. Being an early and sensitive predictor, a fall of ≥50% in CRP at 48 hours can be used to predict the overall response to tocilizumab as a guide to the treatment.

Further, large-scale, well-controlled, randomized studies are needed to determine the protocol of an alternate or escalated treatment in unresponsive patients to tocilizumab as well as assess the treatment response to it.
